# Statewide Community-based Health Promotion: A North Carolina Model to Build Local Capacity for Chronic Disease Prevention

**Published:** 2005-10-15

**Authors:** Marcus Plescia, Suzanna Young, Rosemary L Ritzman

**Affiliations:** North Carolina Division of Public Health, Chronic Disease and Injury Section; The author is also affiliated with the University of North Carolina Department of Family Medicine, Chapel Hill, NC; North Carolina Division of Public Health, Raleigh, NC; North Carolina Division of Public Health, Raleigh, NC

## Abstract

**Background:**

Public health faces major challenges to building state and local infrastructure with the capacity to address the underlying causes of chronic disease. We describe a structured statewide approach to providing technical assistance for local communities to support and develop health promotion capacity.

**Context:**

Over the last two decades, the North Carolina Statewide Health Promotion program has supported local approaches to the prevention and control of chronic disease. In 1999, a major change in the program required local health departments to focus on policy-change and environmental-change strategies for addressing three major risk factors: physical inactivity, poor diet, and tobacco use.

**Methods:**

State program consultants provided technical assistance and training opportunities to local programs on effective policy-change and environmental-change strategies and interventions, based on needs defined by a statewide monitoring and evaluation system.

**Consequences:**

The percentage of health departments in North Carolina with interventions addressing at least one of three targeted risk factors in 2004 approached 100%; in 2001, this percentage was 62%. Additionally, between 2001 and 2004, the number of health departments reporting policy or environmental outcomes related to these risk factors almost doubled.

**Interpretation:**

Requiring local programs to implement policy-change and environmental-change interventions that address the three major behavioral risk factors provides an organized framework for accountability. An established reporting system guides technical assistance efforts and monitors their effectiveness based on standardized objectives that address the full scope of the socioecologic model.

## Background

Public health and medical care systems throughout the United States struggle to address the increasing burden of chronic disease at the national, state, and local levels. Preventable chronic disease conditions represent the nation's leading causes of death and account for 75% of all health care costs (1). Analyses of risk factors for chronic diseases clearly indicate that physical inactivity, poor diet, and tobacco use are the underlying causes of the majority of all deaths each year (2,3). Although chronic diseases have surpassed infectious diseases as the main cause of death and disability in the United States during the last 50 years, local and state public health efforts continue to focus on infectious disease control (4).

A socioecologic approach to community health recognizes that health behaviors are multifaceted and are part of a larger social system of behaviors and social influences. Changes in health behaviors require supportive changes within the following five levels of influence: intrapersonal factors, interpersonal processes and groups, institutional factors, community factors, and public policy (5). Policy changes include changes to laws, regulations, and both formal and informal rules and practice standards. These policy changes lead most often to further changes in the physical and social environment that provide new or enhanced support for positive health behaviors. Programs that target supportive changes at the community, institutional, and policy levels are now encouraged by the public health community as highly effective evidence-based approaches (6). An example of how a community health promotion program would target these intervention levels to promote physical activity might include 1) advocating for county subdivision ordinances and land-use plans to require sidewalks; 2) working with local businesses to provide on-site exercise opportunities for employees; and 3) using a variety of media-based prompts to encourage use of available resources.

Public health faces a major challenge to building state and local infrastructure with the capacity to address the primary risk factors for chronic disease. Despite awareness of the benefits of a more comprehensive approach to community health promotion, implementing policy and environmental change is a difficult process, and there are few reports of states that have made this transition at the community level (7). Strong comprehensive approaches have been made in some state tobacco control programs; much of this success appears to be based on significant resources made available from Master Tobacco Settlement Agreement revenues and on an increasingly well-developed evidence base describing effective policy and environmental interventions for tobacco control (8).

In this paper, we describe a structured statewide approach to providing technical assistance for local communities to support and develop health promotion capacity. This approach is focused on policy-level and environmental-level community-based interventions and includes an evaluation system to monitor progress and guide technical assistance for local communities. This program in North Carolina is oriented toward county health departments, but applications of this model can be adapted for use in a more regionalized public health system.

## Context

The county is the main unit of local government in North Carolina. In 2003, individual county populations ranged from 4226 to 750,221 residents (9). North Carolina has a strong local system of autonomous county health departments that provide the core infrastructure of the state's public health system. Each health department is administered by a health director who is hired and supervised by a local board of health. The state provides pass-through and contracted funding to local health departments from a range of state and federal sources. The state is currently working to design and institute an accreditation system that would help standardize the scope and quality of services provided by local health departments.

In 1985, a North Carolina Legislative Research Study Commission was authorized to study "innovative approaches to finance health promotion and disease prevention efforts in the state." In 1986, the commission's study committee recommended that the legislature create a statewide program to provide resources to local health departments to develop and implement community-based health promotion interventions. To support this effort, called the North Carolina Statewide Health Promotion Program, an annual appropriation of $750,000 was provided in 1987 by the state legislature in addition to a Preventive Health and Health Services (PHHS) Block Grant of $459,461.

The North Carolina Statewide Health Promotion Program provides funding to 85 local health departments and districts to support increased physical activity, healthy eating, and tobacco cessation. During the last two decades, the North Carolina Statewide Health Promotion Program has supported local approaches to the prevention and control of chronic disease in every community across the state. During the initial period of the program, the state provided limited oversight and little program guidance or technical assistance. Health departments used the funds primarily to support adult chronic disease screening and treatment services and patient education programs for high-risk clients. These services were also supported by separate state allocations of Adult Health, Hypertension, and Health Promotion funds (including federal PHHS Block Grants). Annual reports sent to the state documented the number of clients screened and the services provided for each funding source.

In 1999, based on increasing evidence of the effectiveness of community-based policy and organizational approaches to health behavior change, the state reorganized the Statewide Health Promotion Program and changed the program's focus. All state appropriations and federal PHHS Block Grant funding for adult health and primary care services were combined. Local health departments continued to receive a baseline appropriation (approximately $21,000 annually). Based on their prior allocations for adult health funds and hypertension funds, 75 counties were funded above this baseline level.

All health departments were required to prepare 3-year strategic plans to transition toward programs focused on policy and organizational changes to increase physical activity, improve eating habits, and reduce tobacco use. During the transition period, all local health departments were required to use their baseline funding and at least 75% of any above-baseline funds for policy-change and environmental-change strategies. Local programs were also required to participate in a comprehensive monitoring system. These new requirements were implemented as part of the contractual agreement between the state and local health departments that allows individual programs to stipulate performance requirements as addenda to the state's consolidated contract. During the 2004–2005 fiscal year, the program provided $2.7 million to local health departments from PHHS Block Grant funds. State appropriations comprised an additional $1 million.

## Methods

Three regional program consultants provide technical assistance and training to each county on community-based prevention programs and monitor each local program's progress annually. Local health departments designate a health promotion coordinator to serve as the primary liaison to the state program. The local health promotion coordinator is responsible for submitting an annual community action and budget plan that specifies policy-change and environmental-change objectives that address at least one of the targeted risk factors. The action steps in each plan must include the names and roles of community partners. Community action and budget plans are reviewed annually by regional program consultants for approval. Contracts require local health departments to create and maintain local partnerships, work collaboratively with community coalitions to plan and implement health promotion activities, submit plans and reports electronically, and attend regional meetings and approved training programs at least twice yearly.

The North Carolina Statewide Health Promotion Program uses the Progress Check system to document and monitor local activities and outcomes. Progress Check is based on a structured framework developed to evaluate community efforts to prevent cardiovascular disease (10). Local staff members document activities linked to their annual community action plan objectives using an application based on Microsoft Access (Microsoft Corp, Redmond, Wash). Twelve categories are used to describe events; these categories are grouped into three main areas: 1) groundwork, which includes assessment, partnering, planning products, and training; 2) actions, which includes policy-change and environmental-change advocacy, services provided, capacity building, and actions related to working on a regional level to implement programs; and 3) accomplishments, which includes media coverage, resources generated, policy-change outcomes, and environmental-change outcomes.

Local staff members describe a significant activity event and categorize the event based on one or more of the 12 areas described above. The policy and environmental activities reported through the Progress Check system allow regional consultants to monitor each county's progress toward completing its annual action and budget plan and determine the need for technical assistance and training to improve local strategies for change. Progress Check data are exported to state staff for tracking and analysis of progress within individual programs and across the state. Data can be combined across multiple categorical programs (e.g., state diabetes and cardiovascular disease programs), and local programs have the capacity to generate an automated report of their activities.

A program evaluator maintains the Progress Check evaluation system and provides training to local programs. Regional program consultants review local reports to validate entries and identify needs for technical assistance and training.

Activities reported by local programs are related to the county's community action plan and may include events that indicate that objectives were met, partially met, or exceeded. Fortuitous outcomes not connected to original objectives can also be captured by the system. Table 1 summarizes Progress Check data fields for risk factors, age, race and ethnicity, setting, funding source, and collaborating agencies.

An example of a reported policy-change and environmental-change outcome was a county health department's partnership with the county school system to implement the *Take 10!* program. This program integrated daily physical activity opportunities within the academic curriculum for 763 elementary school-aged children in four schools (11). The project coordinator reported event descriptions of the major change activities. The final school-policy changes to implement the Take 10! program and create an additional 26,423 10-minute exercise opportunities for students were reported as environmental and policy outcomes. These activities and outcomes were categorized by risk factor (physical activity), target audience (students in kindergarten through fifth grade), partners (North Carolina Department of Public Instruction, individual school staff), and funding source (Statewide Health Promotion Program and county funds). Additional long-term objectives for the intervention include increased numbers of students with measurements within the recommended body mass index category.

## Consequences

Data collected during program year 2000–2001 were used as a baseline to assess the effectiveness of the transition of local programs to community-based programs by 2003–2004. The reporting system used during 2000–2001 was a simple paper reporting system that did not include all of the information that the Progress Check system captures. Despite limitations of the early reporting system, variables common to both systems demonstrate that the Statewide Health Promotion Program clearly influenced local health promotion activity in three priority areas: targeted risk factors, high-risk populations, and policy and environmental change.

Table 2 compares the number of health departments reporting policy-change or environmental-change outcomes during program years 2001–2002 and 2003–2004. The percentage of health departments in North Carolina with interventions addressing at least one of three targeted risk factors during 2003–2004 approached 100%, with almost three quarters reporting activities addressing all three risk factors at the policy-change or environmental-change level or both. These data contrast dramatically with baseline data collected during 2000–2001, indicating that about 40% of health departments addressed physical activity, 32% addressed nutrition risk factors, 56% addressed tobacco use, and only 20% addressed all three. There was a similar increase during the 3-year period in the number of health departments reporting policy or environmental outcomes related to these risk factors. For 2001–2002, 62% of local programs reported policy-change or environmental-change outcomes. For 2003–2004, 93% reported policy-change or environmental-change outcomes. During the same period, outreach to targeted minority populations increased from 18% to 74%. Implementation of programs in community, school, faith, and worksite settings increased dramatically.

Examples of specific outcomes documented by local programs during the 2003–2004 program year include the following:

- 40 school districts in North Carolina established 100% smoke-free campuses (an increase from only 15 of 115 school districts in North Carolina before 2003–2004).
- 41 county school systems in North Carolina implemented healthy meal and snack options for schoolchildren.
- 14 counties in North Carolina increased walking and bike-riding trails by more than 41 miles in their communities.
- 36 counties in North Carolina partnered with work sites to implement policies and facilities that support employees in increasing their physical activity levels and access to healthy eating options.

Although state and federal funding for the Statewide Health Promotion Program is limited — approximately one dollar per North Carolina resident is allocated —  local health departments and their community partners have used these funds to leverage additional local funding. During the state fiscal year 2003–2004, more than $5 million in local and private resources were generated from the $3.7 million state allocation to local programs.

## Interpretation

Increased capacity must be developed at the federal, state, and local levels to affect the rates of chronic disease in the United States. Noncategorical funding for state and local health promotion efforts, however, has received recent criticism and faces significant budget reductions because of concerns that such resources are not used in a standardized or evidence-based way. Our experience in establishing a statewide health promotion program addresses these concerns. Requiring local programs to implement policy-change and environmental-change interventions that address the three major behavioral risk factors provides an organized framework for accountability. The reporting system we established allows state staff members to monitor the effectiveness of local programs in achieving their objectives, provides a basis for tailoring technical assistance to a county's specific needs, and creates a mechanism for performance-based allocations of limited health promotion resources.

A comprehensive reporting system makes it possible to document statewide policy and environmental changes addressing chronic disease prevention. These data, however, have some limitations. Because the program framework allows local health departments to write county-specific objectives that might include a variety of outcomes, it is difficult to summarize statewide changes in a particular area of interest, such as policies on school nutrition or community opportunities for physical activity. The outcomes documented in the Progress Check system are useful for assessing process changes. They do not address overall indices of community change within a particular county or at the state level. The impact on local objectives is also difficult to assess and compare among counties because their definitions of policy-change and environmental-change outcomes can range from minimal to significant.

Several lessons were learned from the development of this statewide approach to health promotion. Funding was initially provided to local health departments without consistent central guidance and oversight. Local agencies allocated resources based on their agency's priorities and funding needs; they resisted the introduction of specific performance expectations. Many local program coordinators have professional backgrounds in working with individuals; they struggled with the change in program guidelines. The initiation of a structured approach to program accountability has provided a basis for instituting performance-based funding allocations. The state can now use Progress Check and program monitoring to decrease local allocations based on poor performance and reallocate resources to counties with high performance levels. Funding has been reduced to some noncompliant health departments. For example, when recent state and federal program funding reductions occurred, the Statewide Health Promotion Program used performance measures to implement cuts rather than reduce all counties equally as it had done in the past. There are limits to this approach, however, because of the politics of state government. Attempts are underway in North Carolina to institute a local health department accreditation system with additional provisions for performance-based funding. This would provide better mechanisms for enforcing program guidelines and would allow the development of links between local plans and statewide outcomes.

Introducing new technology and reporting requirements for local programs also required planning at the state level to ensure that training and technical assistance for local programs was accessible at the time the changes occurred. Initially, many local health promotion staff had limited computer skills and felt intimidated by the reporting system. Individual training, ongoing technical consultation, and reassurance from the consultants were necessary to resolve these issues. Providing local programs the capacity to generate their own reports proved to be one of the most critical factors in increasing local acceptance of the monitoring system and improving the quality and consistency of data reported to the state. Local programs could also use the summary data to prepare reports to local government agencies and develop grant proposals for additional resources.

Requiring local health departments to transition health promotion funding to community-based interventions rather than clinical services could raise concerns that screening and adult health services may not be available in these communities. In North Carolina, the transition of health promotion funds to community-based programs was part of a trend by many local health departments to discontinue primary care services. Communicable disease services and other essential public health services remain, but adult health, home health, and in many locations, prenatal and child health services have been transferred to local hospitals and community health centers. This transfer has allowed health departments to focus limited resources on community-based public health programs and services.

Health promotion interventions are often most effective when implemented at the community level. The North Carolina Statewide Health Promotion Program is a structured model for evidence-based approaches and the development of local capacity for health promotion practice that can be used by other states. Although North Carolina has been successful in obtaining federal categorical funding for chronic disease programs, the Statewide Health Promotion Program is funded by federal block grant funds (PHHS Block Grants) and moderate levels of state funding. The program could be adopted by other states using similar resources. The Progress Check monitoring system is adaptable; technical assistance is provided by a limited number of staff. Other states could face similar challenges in developing local acceptance of a more structured framework and commitment to consistent standards. States with a more regionalized, district-oriented infrastructure or a well-developed system of local accreditation would be particularly well prepared to institute this system.

## Figures and Tables

**Table 1:** Table 1. Data Fields, Computer-based Progress Check System for Documenting and Monitoring Local Activities and Outcomes, North Carolina Statewide Health Promotion Program

**Risk Factor Addressed**	**Population Age**	**Population Race and Ethnicity**	**Setting**	**Funding Source**	**Collaborating Agency**
Nutrition Physical activity Tobacco Overweight or obesity Other	General population School and youth Preschooler Infant and toddler Senior	General population African American Hispanic American Indian Asian	Community Schools or childcare Faith community Worksite Community group Health care institution	State health department Local Nonprofit Private for-profit Other	Business Chamber of Commerce College or university Community group Cooperative extension Faith community Health department Hospital Local parks and recreation organization Media Nonprofit organization Planning office School Transportation office Other state agency Community health center Faith-supported clinic

**Table 2:** Reported Local Health Department Events, by Outcomes, Risk Factors, Race and Ethnicity, and Settings, North Carolina, 2001–2002 and 2003–2004

**Event Characteristics**	**Health Departments Reporting^a^**			

	**2000-2001** **(N = 78)**		**2003-2004** **(N = 85)**	

	**No. **	**% **	**No. **	**% **

**Outcomes**				

Environmental/policy change	48	61.5	79	92.9

**Risk factors**				

Physical activity	31	39.7	85	100.0
Nutrition	25	32.1	84	98.8
Tobacco	44	56.4	77	90.6
All	16	20.5	62	72.9

**Race and ethnicity**				

General population	66	84.6	85	100.0
Racial and ethnic minority populations	14	17.9	63	74.1

**Settings**				

Community	26	33.3	84	98.8
Schools	30	38.5	85	100.0
Faith organizations	9	11.5	58	68.2
Worksites	10	12.8	80	94.1

a The total number of local health departments in North Carolina is 85.
